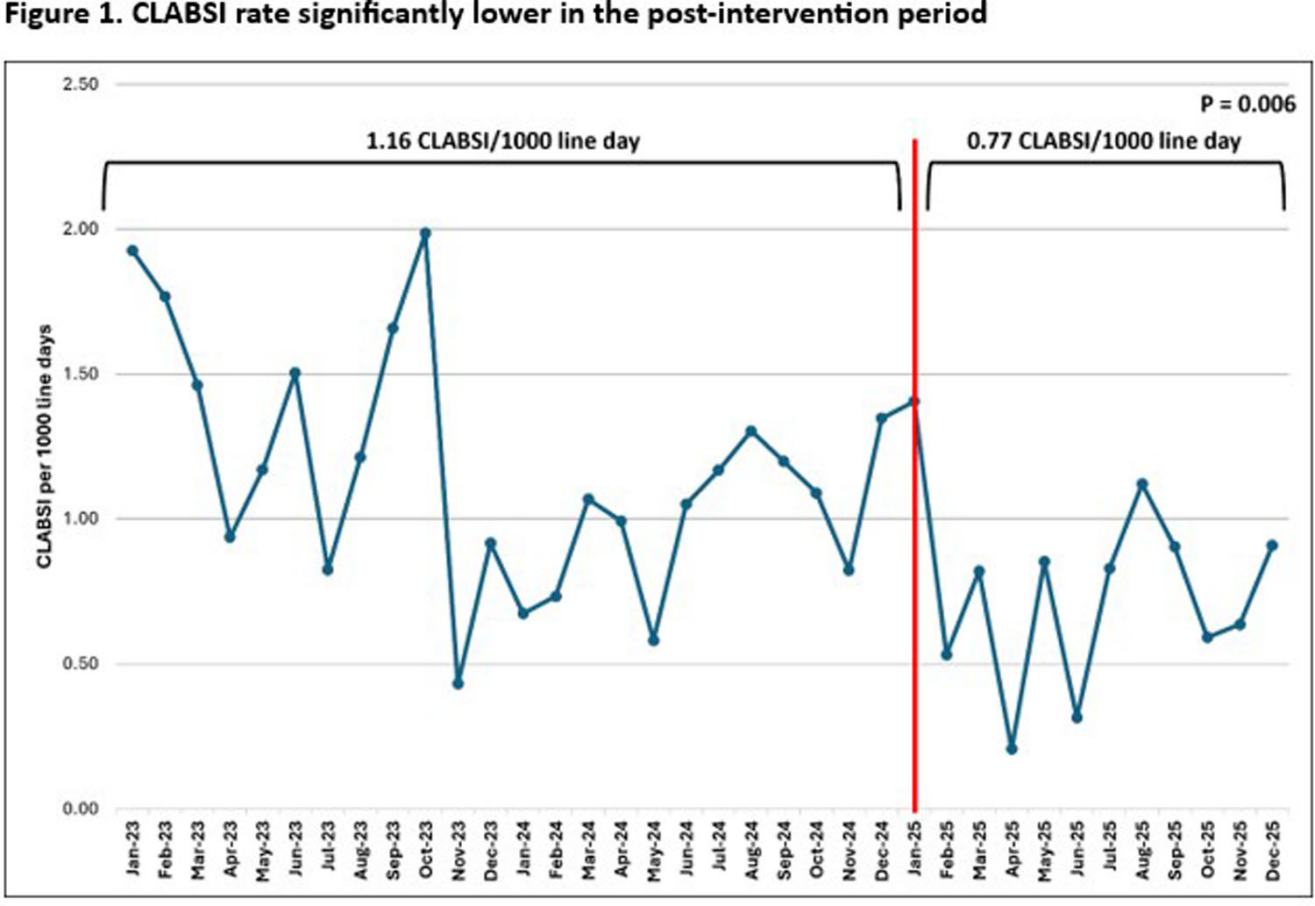# 353 From Colonization to Prevention: Reducing Staphylococcus aureus Burden with Decolonization

**DOI:** 10.1017/ash.2026.10455

**Published:** 2026-06-23

**Authors:** Brandi Manning, Shandra Day, Satomi Abe, Kelci Coe, Brandy Gilbert, Holly Chignoli, Heather Smith, Sydney Agnello, Nora Colburn

**Affiliations:** 1 The Ohio State University; 2 Ohio State University; 3 The Ohio State University Wexner Medical Center; 4 Ohio State Wexner Medical Center; 5 The Ohio State Wexner Medical Center

## Abstract

**Background:** Blood culture contamination leads to unnecessary clinical interventions and increased healthcare costs. Estimations indicate each contamination incurs over $4,500 in avoidable cost. The 2024 IDSA/ASM guideline advises blood culture collection via peripheral venipuncture and cultures from existing vascular catheters should be reserved for suspected Central line associated bloodstream infection (CLABSI) and paired with a peripheral set. It was observed that routine ordering and collection of blood cultures from central venous catheters (CVC) was common practice at our institution and our blood culture contamination rate was above the recommended 1% goal. A multidisciplinary quality improvement plan was implemented to reduce inappropriate catheter blood culture collection. **Methods:** A pre-post intervention study was conducted at a large quaternary medical center from January 1, 2023 to December 31, 2024 (pre-intervention) and January 1, 2025 to December 31, 2025 (post-intervention). The intervention included simultaneous updates to 1) provider clinical practice guidelines for blood culture indications, 2) nursing policies on culture collection techniques and 3) nutrition policies regarding surveillance cultures from CVCs prior to initiation of total parenteral nutrition. Education was provided and reinforced at multidisciplinary quality rounds as well as various meetings, huddles and lectures. Blood culture contamination was defined using CLSI criteria and rates and collection sites were compared pre and post intervention. CLABSI was determined using National Healthcare Safety Network (NHSN) definitions. CLABSI rates and blood culture collection sites were compared pre and post-intervention. **Results:** During the study period, 185,030 sets of blood cultures were collected. The number of blood cultures collected from a CVC significantly decreased (Table 1) and the contamination rates of cultures significantly decreased from both peripheral venipuncture and CVC. By reducing collection from lines, 29 estimated contaminations were avoided, with an associated cost savings of $131,602 and a reduction of 203 antimicrobial days. There was a significant decrease in CLABSI rate in the post-intervention period (Figure 1). There were significantly fewer blood cultures drawn from CVCs in patients with NHSN CLABSI in the post-intervention period (70% vs 50%, p = 0.0005). **Conclusions:** The simultaneous policy change and multifaceted reinforcement with strong senior leadership support resulted in rapid adoption and sustained change in blood culture ordering and collection practices. There was a significant decrease in the proportion of blood cultures drawn from CVCs, blood culture contamination and CLABSI rate in the post-intervention period leading to significant cost savings, fewer antimicrobial days and improved patient outcomes.

**Figure:**
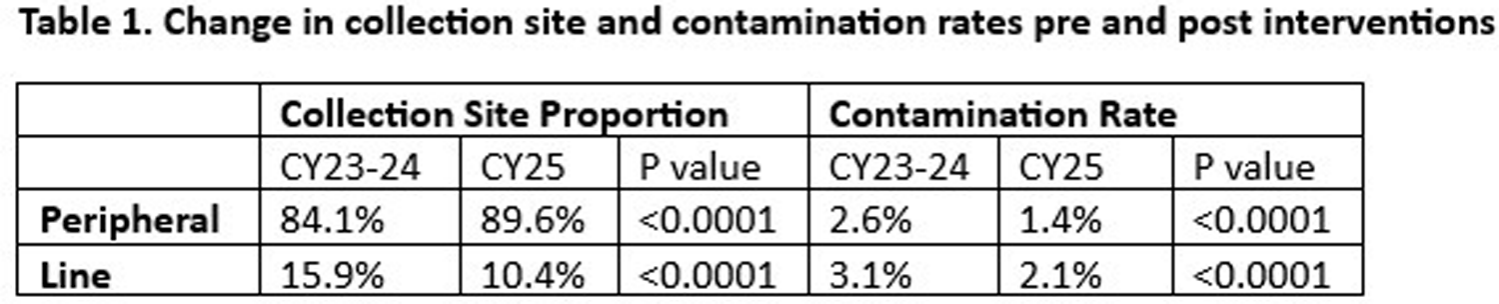

**Figure:**